# *Ts*-Hsp70 induces protective immunity against *Trichinella spiralis* infection in mouse by activating dendritic cells through TLR2 and TLR4

**DOI:** 10.1371/journal.pntd.0006502

**Published:** 2018-05-18

**Authors:** Rui Zhang, Qing Sun, Yi Chen, Ximeng Sun, Yuan Gu, Zhang Zhao, Yuli Cheng, Limei Zhao, Jingjing Huang, Bin Zhan, Xinping Zhu

**Affiliations:** 1 Department of Medical Microbiology and Parasitology, School of Basic Medical Sciences, Capital Medical University, Beijing, China; 2 Research Centre of Microbiome, Capital Medical University, Beijing, China; 3 Department of Pediatrics, National School of Tropical Medicine, Baylor College of Medicine, Houston, Texas, United States of America; Queen's University Belfast, UNITED KINGDOM

## Abstract

**Background:**

Trichinellosis is a serious food-borne parasitic zoonosis worldwide. In the effort to develop vaccine against *Trichinella* infection, we have identified *Trichinella spiralis* Heat shock protein 70 (*Ts*-Hsp70) elicits partial protective immunity against *T*. *spiralis* infection via activating dendritic cells (DCs) in our previous study. This study aims to investigate whether DCs were activated by *Ts*-Hsp70 through TLR2 and/or TLR4 pathways.

**Methods and findings:**

After blocking with anti-TLR2 and TLR4 antibodies, the binding of *Ts*-Hsp70 to DCs was significantly reduced. The reduced binding effects were also found in TLR2 and TLR4 knockout (TLR2^-/-^ and TLR4^-/-^) DCs. The expression of TLR2 and TLR4 on DCs was upregulated after treatment with *Ts*-Hsp70 *in vitro*. These results suggest that *Ts*-Hsp70 is able to directly bind to TLR2 and TLR4 on the surface of mouse bone morrow-derived DCs. In addition, the expression of the co-stimulatory molecules (CD80, CD83) on *Ts*-Hsp70-induced DCs was reduced in TLR2^-/-^ and TLR4^-/-^ mice. More evidence showed that *Ts*-Hsp70 reduced its activation on TLR2/4 knockout DCs to subsequently activate the naïve T-cells. Furthermore, *Ts*-Hsp70 elicited protective immunity against *T*. *spiralis* infection was reduced in TLR2^-/-^ and TLR4^-/-^ mice correlating with the reduced humoral and cellular immune responses.

**Conclusion:**

This study demonstrates that *Ts*-Hsp70 activates DCs through TLR2 and TLR4, and TLR2 and TLR4 play important roles in *Ts*-Hsp70-induced DCs activation and immune responses.

## Author summary

Trichinellosis is a serious food-borne parasitic zoonosis caused by tissue-dwelling nematode *Trichinella spiralis*. Vaccine development is needed as an alternative approach to control the infection in domestic livestock or in humans. *Ts*-Hsp70 has been identified to elicit partial protective immunity against *Trichinella spiralis* infection via activating dendritic cells (DCs) in our previous study. This study aims to investigate the pathway(s) through which the *Ts*-Hsp70 activates DCs. Our results identified that *Ts*-Hsp70 could bind to DCs which was inhibited by blocking TLR2 and TLR4 with antibodies or TLR2 and TLR4 knockout. *Ts*-Hsp70 stimulated the expression of TLR2 and TLR4 and the co-stimulatory CD80, CD83 and CD86 on the surface of DCs which was reduced in TLR2 or TLR4 knockout mice. With TLR2 or TLR4 knockout, DCs were less stimulated by *Ts*-Hsp70 and subsequently reduce the activation of naïve T-cells. The protective immunity induced by *Ts*-Hsp70 against *T*. *spiralis* infection was also reduced in TLR2 or TLR4 knockout mice. The results conclude that *Ts*-Hsp70 activates DCs through activating TLR2 and TLR4 and TLR2 and TLR4 play important roles in *Ts*-Hsp70-induced protective immunity against *Trichinella* infection.

## Introduction

Trichinellosis is a serious food-borne parasitic zoonosis caused by eating raw or undercooked meats contaminated with larvae of *Trichinella spiralis* [1, 2]. There are about 11 million people infected with this nematode worldwide [3]. Heavy infection causes serious muscle pain and other complications or even death [4]. Due to the wide distribution of wild and domestic animals infected with *T*. *spiralis* as sources of infection and the difficult diagnosis of the infection because of the non-specific symptoms, trichinellosis is still not under control in endemic areas and vaccine development is needed as an alternative approach to prevent the infection in domestic livestock or in humans [5–7].

Heat shock proteins (Hsps), a family of highly conservative stress proteins, are produced under different pressure conditions such as heat shock, oxygen radicals, nutrient deprivation and metabolic disruption [8]. Some Hsps have been reported to play important roles in antigen presentation and maturation of dendritic cells [9, 10]. Recently, many studies have showed that Hsps from parasites [11, 12] or bacteria [13] exhibited potent immunogenicity and induced protective immunity against specific infections, thus these proteins have become momentous target proteins in vaccine development against various infections. Heat shock protein-70 of *Trichinella spiralis* (*Ts*-Hsp70) is a member of Hsp70 family with molecular weight of about 70 kDa and an immunodominant antigen during infection. *Ts*-Hsp70 has been proved to be a good vaccine candidate against *T*. *spiralis* infection, mice immunized with *E*. *coli* expressed recombinant *Ts*-Hsp70 (r*Ts*-Hsp70) formulated with Freund’s adjuvant produced 37% muscle larvae reduction compared with control mice [14]. The protective immunity induced by immunization of r*Ts*-Hsp70 was related to the stimulation of host dendritic cells (DCs). Incubation of mouse bone marrow-derived DCs with r*Ts*-Hsp70 leaded to the maturation of DCs characterized by the increased surface expression of CD11c, MHC II, CD40, CD80, and CD86 and the secretion of IL-1β, IL-12p70, TNF-α, and IL-6. The r*Ts*-Hsp70-stimulated DCs enabled to activate CD4^+^ T cell and prime a protective immunity in mice against *T*. *spiralis* infection [15]. However, the molecular mechanism and the activation pathway of r*Ts*-Hsp70 priming DCs are not well understood.

It is well known that antigens from some pathogens mainly activate DCs *via* pattern recognition receptors (PRRs) signaling pathway [16–18]. PRRs play a key role in host cell recognition and response to microbial pathogens [19–21]. Since DCs is an important antigen-presenting cell (APC), many types of PRRs are expressed on the surface of DCs to identify and distinguish different pathogens related antigen [22]. Among these PRRs, toll-like receptors (TLRs) are the most important members expressed on the surface of DCs. Mammalian TLRs consist of 13 members, and TLR4 is the first member discovered and has been proved to induce the activation and expression of NF-κB, which controls the genes for the inflammatory cytokines [23]. Recent researches have showed that the specific immune responses caused by helminth infections were closely related with TLRs, and TLR2 and TLR4 are most frequently involved [24–26]. For example, the excretory–secretory (ES) antigens from *Schistosoma sp*. bound and activated DCs through TLR2 [27]; the excretory–secretory products 62 (ES-62) from *Acanthocheilonema viteae* activated DCs through TLR4 and induced Th2 immune response [28]. In this study, we investigated whether r*Ts*-Hsp70 activated DCs via TLR2 or TLR4, and what role the TLR2 and TLR4 played in the protective immunity induced by immunization with r*Ts*-Hsp70 against *T*. *spiralis* infection.

## Materials and methods

### Animals

All animal experiments were approved by the Capital Medical University Animal Care and Use Committee on the Ethics of Animal Experiments (Permission No. AEEI-2015-136) and were in accordance with the NIH Guidelines for the Care and Use of Laboratory Animals. Female C57 BL/6 wild-type (WT) mice with 6–7 weeks old were purchased from the Laboratory Animal Services Center of the Capital Medical University (Beijing, China). Female C57 BL/6 TLR2^-/-^ (TLR2 gene knockout) and TLR4^-/-^ (TLR4 gene knockout) mice with the same age were purchased from Nanjing University Biomedical Research Institute (Nanjing, China). All mice were maintained in specific pathogen-free conditions.

### Parasites

*T*. *spiralis* (strain ISS 533) was firstly isolated from a swine in Hei Longjiang, China and maintained in female ICR mice. Muscle larvae (ML) were isolated from the infected mice via the standard pepsin-hydrochloric digestion method for oral challenge test as previously described [29]. Briefly, the muscle tissues of infected mice were cut into pieces and digested by pepsin-hydrochloric digestive fluid. The ML were collected by washing twice in water with sedimentation and counted with gelatin.

### Recombinant *Ts*-Hsp70 protein preparation

Recombinant *Ts*-Hsp70 (r*Ts*-Hsp70) was expressed in *E*. *coli* (BL21) and purified as previously described [14]. The contaminated endotoxin was effectively removed by ToxOut High Capacity Endotoxin Removal Kit (Biovision, USA). The residual endotoxin was 0.1984 EU/mg in the final purified r*Ts*-Hsp70, approximately equivalent to 20 pg/mg endotoxin in r*Ts*-Hsp70 [30], which is lower than the minimal amount that could stimulate TLR2/4 based on the instruction of standard LPS O55: B5 (Invivogen, USA). The r*Ts*-Hsp70 was labeled with phycoerythrin (PE) by Beijing Biosynthesis Biotechnology company, LTD.

### Preparation for mouse bone marrow-derived dendritic cells

DCs were generated from mouse bone marrow according to the method previously described [31]. Briefly, the mouse bone marrow cells were cultured at the density of 1×10^6^ cells/ml in RPMI 1640 medium supplemented with 10% fetal bovine serum (FBS, Gibco, USA), 2% penicillin-streptomycin (Hyclone, USA), 10 ng/ml recombination granulocyte/macrophage colony-stimulating factor (GM-CSF, Pepro Tech, USA) and 1 ng/ml IL-4 (Pepro Tech, USA) at humidified atmosphere at 37°C, 5% CO_2_ for 7 days with semi-quantitative medium change daily. On day 7, the non-adherent and low-adherent cells were harvested as immature DCs (purity was > 50%).

### Detection of r*Ts*-Hsp70 binding to TLR2 and TLR4 on DCs

DCs from WT C57 BL/6 mice were prepared as described above. The immature DCs were collected and washed with PBS. Cell suspension was firstly pre-incubated with IgG1 Isotype antibody (Sigma, USA), TLR2 blocking antibody (Biolegend, USA) or TLR4 blocking antibody (Biolegend, USA) on ice for 1 h. After being washed with PBS, the cells were stained with anti-mouse CD11c APC and anti-TLR2-FITC, anti-TLR4-PE-Cy7, r*Ts*-Hsp70-PE on ice for 30 min. The percentage of r*Ts*-Hsp70 binding to TLR2 or TLR4 on DCs (r*Ts*-Hsp70-PE and TLR2-FITC or TLR4-PE-Cy7 double positive cells from CD11c^+^ cells) was analyzed by Flow Cytometry (BD Biosciences, USA). Dead cells and doublets were excluded from all analysis.

### TLRs expression and detection

WT C57 BL/6 mice bone marrow DCs were prepared as described above and then respectively stimulated with 5 μg/ml of r*Ts*-Hsp70, 50 ng/ml of lipopolysaccharide (LPS, a agonist of TLR4, InvivoGen, USA), 200 ng/ml of Pam_3_CysSerLys_4_ (Pam_3_CSK_4_, a agonist of TLR2, InvivoGen, USA) on day 5 for 48 h. Control cells were added with 20 μl phosphate-buffered saline (PBS) or 5 μg/ml of bull serum albumin (BSA, Thermo, USA). The stimulated DCs were collected and washed with 3 ml PBS once. The collected DCs were firstly pre-incubated with Fc Blocker (Anti-Mouse CD16/CD32, BD Biosciences, USA) for 30 min to reduce non-specific binding of labelled antibodies, then stained with anti-mouse CD11c APC (eBioscience, USA), anti-mouse CD282 (TLR2) FITC (eBioscience, USA) or anti-mouse CD284 (TLR4) PE (eBioscience, USA) on ice for 30 min, respectively. The percentage of TLR2 and TLR4 expressing cells (CD11c-APC and TLR2-FITC/TLR4-PE double positive) was analyzed by Flow Cytometry (BD Biosciences, USA). Doublets were excluded from all analysis.

### Dendritic cell activation and detection

To induce the maturation of DCs, the bone marrow cells from WT, TLR2^-/-^, TLR4^-/-^ mice were cultured as described above and then respectively stimulated with 5 μg/ml of r*Ts*-Hsp70, or 50 ng/ml of LPS or 200 ng/ml of Pam_3_CSK_4_ on day 5 for 48 h. On day 7, the mature DCs were harvested and pre-incubated with Fc Blocker. The mature DCs were then stained with anti-mouse CD11c APC and anti-CD80 PE, or anti-CD83 PE, or anti-CD86 PE (eBioscience, USA) on ice for 30 min. The cells were washed and re-suspended in PBS. The expression of co-stimulatory molecules (CD80, CD83, CD86) on the surface of stimulated DCs were detected by Flow Cytometry (BD Biosciences, USA). Dead cells and doublets were excluded from all analysis. Simultaneously, the culture supernatants were collected for detecting cytokines (IL-1β, IL-4, IL-6 and TNF-α) secreted by these stimulated DCs as described below (ELISA).

### Isolation of naïve CD4^+^ T cells from splenocytes

The spleens were isolated from the normal mice and homogenized as single cells in 6 ml Mouse 1×Lymphocyte Separation Medium (Dakewe biotech, China) in 15 ml tube. The cell suspension was centrifuged to separate the lymphocytes. The splenocytes in the middle layer were removed using a glass pipette, then washed with PBS and re-suspended in RPMI 1640 medium. The CD4^+^ T cells were isolated from the splenocytes using the CD4^+^ T cell isolation kit (Miltenyi Biotec, Germany) with CD4^+^ T cell biotin-antibody cocktail, anti-biotin microbeads and MACS columns (purity was > 95%).

### DCs and lymphocytes co-incubation *in vitro*

DCs from WT, TLR2^-/-^ or TLR4^-/-^ mice were cultured for 5 days and stimulated as described above for 48 h. The stimulated DCs were respectively collected and washed once with RPMI1640, then re-suspended at 1×10^6^ cells/ml in RPMI1640 medium supplemented with 10% fetal bovine serum (FBS, Gibco, USA), 2% penicillin-streptomycin (Hyclone, USA). The CD4^+^ T cells were labeled with 2 mM CFSE (Invitrogen, USA). The CFSE-labeled CD4^+^ T cells were re-suspended at 5×10^6^ cells/ml. The two cell suspensions were co-cultured (DC: T cell = 1:5, 50 μl/50 μl) in the 96-well plates at a humidified atmosphere of 5% CO_2_ at 37°C for 3 days. The CD4^+^ T cells proliferated when stimulated with these activated DCs, resulting in dilution of CFSE content. The CD4^+^ T cell proliferation was monitored by CFSE dilution using a Flow Cytometer (BD Biosciences, USA). Dead cells and doublets were excluded from all analysis.

### Intracellular cytokine staining

For the detection of cytokines, the stimulated DCs (1×10^6^ cells/ml) as described above and splenocytes (1×10^7^ cells/ml) suspensions were co-cultured (1:10, 50 μl/50 μl) in media for 3 days. The cells were incubated with 10 mg/ml Brefeldin A, 50 ng/ml phorbol 12-myristate 13-acetate (PMA) (Sigma) and 750 ng/ml Ionomycin (Sigma) for 6 h at 37°C. Surface staining was performed for 30 min with anti-CD3-APC and anti-CD4-FITC on ice. After surface staining, the cells were resuspended in Fixation/Permeabilization solution (BD, Cytofix/Cytoperm kit), and intracellular cytokine staining was performed using anti-IFN-γ-PE, anti-IL-2-PE, anti-IL-4-PE-Cy7 and anti-IL-6-PE antibodies. The cell suspension was analyzed by Flow Cytometry (BD Biosciences, USA). Doublets were excluded from all analysis.

### Immunization with r*Ts*-Hsp70 and larvae challenge

Groups of WT, TLR2^-/-^, TLR4^-/-^ C57 BL/6 mice (each 20 mice) were subcutaneously immunized with 30 μg r*Ts*-Hsp70 without adjuvant for three times at two weeks interval. One group of mouse (20 mice each) was each given PBS only as control. One week after last immunization, 10 mice from each group were sacrificed for collecting sera and splenocytes for immunogenicity test as described below, the left 10 mice from each group were each orally challenged with 500 infective *T*. *spiralis* larvae. Muscle larvae were harvested and counted 45 days post-infection as described above. Muscle larvae burden reductions in immunized mice were evaluated according to the following formula:

$$\mathrm{larvae}\;\mathrm{reduction}\;\%=(1‑\frac{\mathrm{mean}\;\mathrm{number}\;\mathrm{of}\;\mathrm{larvae}\;\mathrm{per}\;\mathrm{gram}\;\mathrm{muscle}\;\mathrm{in}\;\mathrm{immunized}\;\mathrm{mice}}{\mathrm{mean}\;\mathrm{number}\;\mathrm{of}\;\mathrm{larvae}\;\mathrm{per}\;\mathrm{gram}\;\mathrm{muscle}\;\mathrm{in}\;\mathrm{control}\;\mathrm{mice}})\times 100\%$$

### Proliferation of splenocytes from immunized mice

One week after the last immunization, the splenocytes from each immunized mice was isolated as described above. The splenocytes were suspended at 2×10^6^ cells/ml in the 96-well plates, then re-stimulated with r*Ts*-Hsp70 at 5 μg/ml for 3 or 4 days. The proliferation of the splenocytes was determined by MTS colorimetric assay (Promega, USA) and the re-stimulated culture supernatants were collected for detecting cytokine (IFN-γ, IL-2, IL-4 and IL-6) secretion. Proliferation index of splenocytes from immunized mice was evaluated according to the following formula:

$$\mathrm{Proliferation}\;\mathrm{index}=\frac{\left({\mathrm{OD}}_{\mathrm{Hsp}70}-{\mathrm{OD}}_{1640}\right)-\left({\mathrm{OD}}_{\mathrm{PBS}}-{\mathrm{OD}}_{1640}\right)}{\left({\mathrm{OD}}_{\mathrm{PBS}}-{\mathrm{OD}}_{1640}\right)}$$

### Serological antibody detection

The serum was collected from each immunized mouse one week after the final immunization. The titer of anti-r*Ts*-Hsp70-specific IgG, IgG1 and IgG2a antibody in the sera of immunized mice was determined by ELISA as previously described [32]. Briefly, 96-well plates were coated with r*Ts*-Hsp70. After being blocked with 5% BSA (in PBS), serum samples at doubling dilution in PBS were added into the plate and incubated for 1 h. HRP-conjugated goat anti-mouse IgG, IgG1, IgG2a (BD pharmingen, USA) was used as secondary antibodies. The o-phenylenediamine dihydrochloride substrate (OPD, Sigma, USA) was added to visualize the results. The reaction was stopped by adding 20% H_2_SO_4_. The OD was detected at 492nm. The endpoint titer of immune sera was determined by the final dilution with OD ≥ 2.1 over control mouse sera (PBS group).

### Cytokine detection

The levels of IL-4, IL-6, IL-1β and TNF-α were measured by ELISA kits (Dakewe biotech, China). All the process was carried out according to manufacturer’s instruction.

### Statistical analysis

The data were shown as the mean ± the standard deviation (S.D.). All data were compared by analysis of variance (one-way ANOVA) and Student’s *t*-test using GraphPad Prism 5 (GraphPad Software, USA). *p* < 0.05 was considered as statistically significant.

## Results

### r*Ts*-Hsp70 binds to TLR2 and TLR4 on DCs *in vitro*

**Fig 1:**
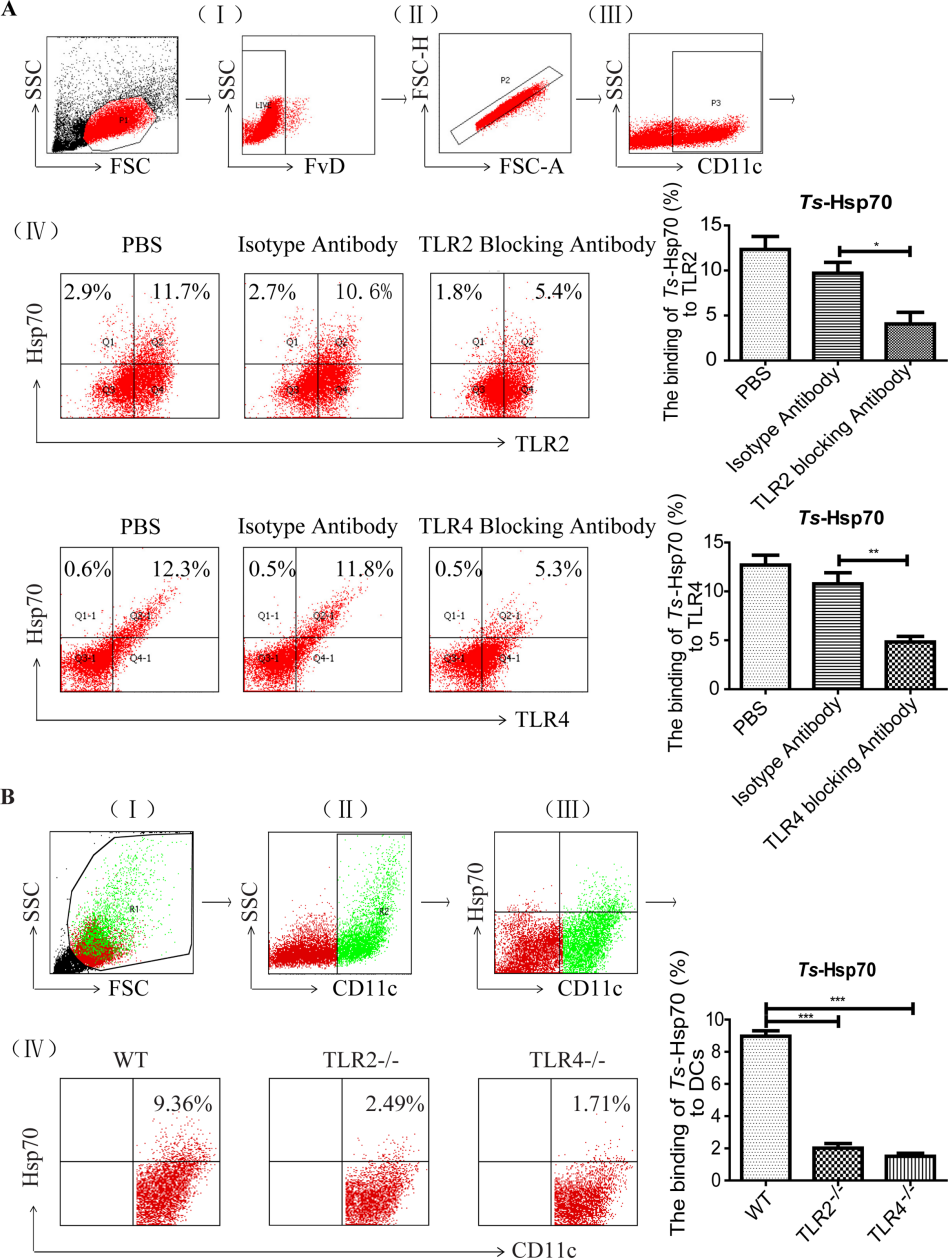
r*Ts*-Hsp70 binds to DCs through TLR2 and TLR4 *in vitro* detected by flow cytometry. (A) Representative dot plots for the gating strategy: (I) gating on viable cells, (II) selection of non-adherent cells, (III) gating on CD11c^+^ cells, and (IV) selection of TLR2^+^ and Hsp70^+^ from gated CD11c^+^ cells (upper panel) and TLR4^+^ and Hsp70^+^ from gated CD11c^+^ cells (lower panel), respectively. (B) The binding of r*Ts*-Hsp70 to DCs derived from WT, TLR2^-/-^ or TLR4^-/-^ mice *in vitro*. DCs derived from WT, TLR2^-/-^ or TLR4^-/-^ mice were stained with anti-mouse CD11c APC and r*Ts*-Hsp70-PE. Representative dot plots for the gating strategy: (I) gating on viable cells, (II) gating on CD11c^+^ cells, (III) selection of Hsp70^+^ and CD11c^+^ from gated R1 cells, and (IV) selection of r*Ts*-Hsp70^+^ from gated CD11c^+^ cells. The percentage of CD11c+ cells binding to r*Ts*-Hsp70 shown on the right. All experiments were performed three times and data are shown with mean ± SD. n = 3, * *p* < 0.05, ** *p* < 0.01, *** *p* < 0.001.

To determine if r*Ts*-Hsp70 binds to DCs *in vitro*, PE-labeled r*Ts*-Hsp70 was used to stain the DCs, and the stained cells were analyzed by flow cytometry. As shown in Fig 1A, total 11.7% TLR2^+^ and 12.3% TLR4^+^ on CD11c^+^ DCs were bound by r*Ts*-Hsp70. After being blocked with either TLR2 or TLR4 antibodies, the percentage of TLR2/4 binding to r*Ts*-Hsp70 in DCs was significantly decreased compared to those cells incubated with isotype antibody control, indicating r*Ts*-Hsp70 binds to DCs through TLR2 and TLR4. The bone marrow-derived DCs from TLR2 knockout mice (TLR2^-/-^) or TLR4 knockout mice (TLR4^-/-^) also showed significant lower binding activity with r*Ts*-Hsp70 (Fig 1B), further confirming that r*Ts*-Hsp70 binds to DCs through both TLR2 and TLR4.

### r*Ts*-Hsp70 upregulates the expression of TLR2 and TLR4 on DCs *in vitro*

**Fig 2:**
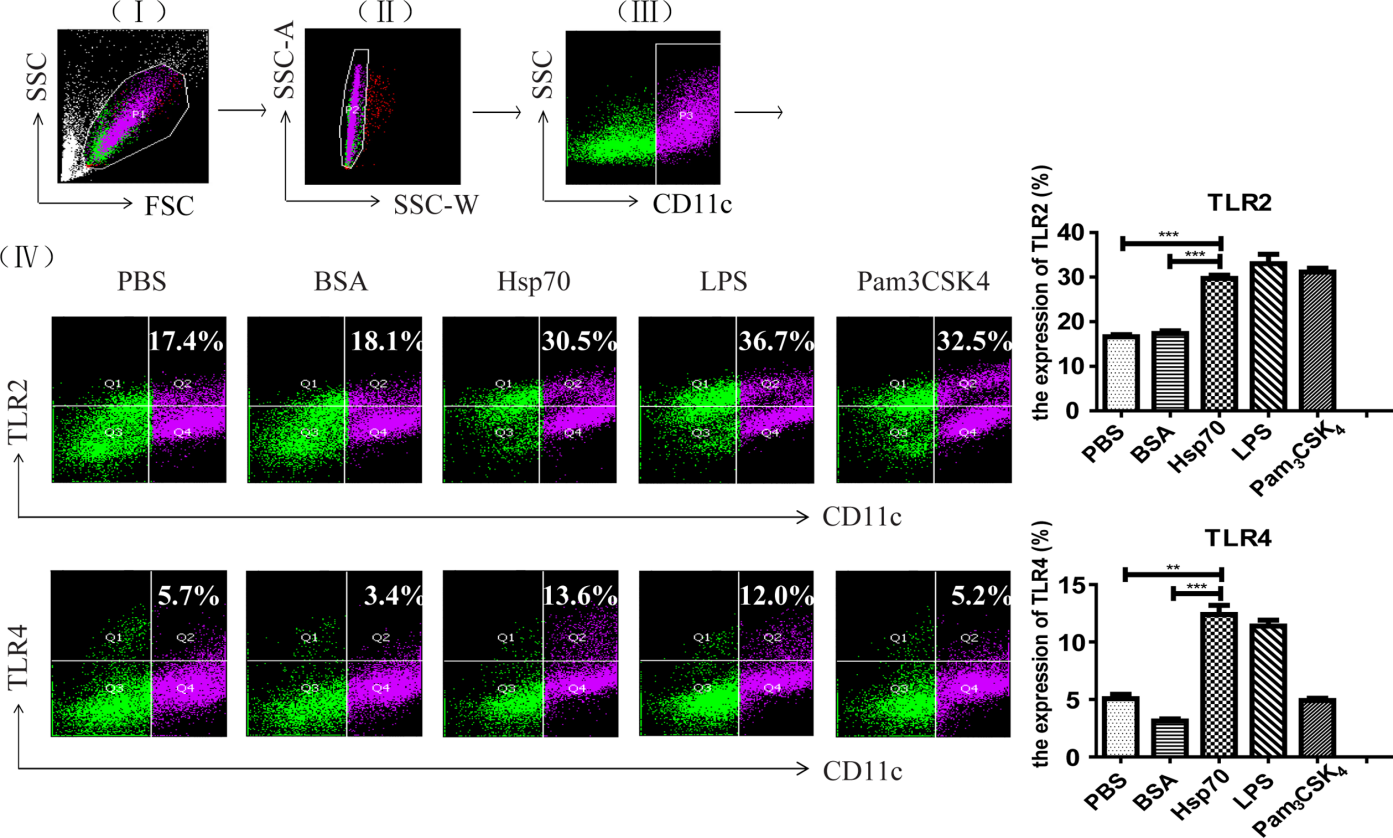
Upregulation of both TLR2 and TLR4 on the surface of mouse bone marrow-derived DCs stimulated by r*Ts*-Hsp70. Representative dot plots for the gating strategy: (I) gating on viable cells, (II) selection of non-adherent cells, (III) gating on CD11c^+^ cells, and (IV) selection of TLR2^+^ cells from gated CD11c^+^ cells (upper panel) and TLR4^+^ cells from gated CD11c^+^ cells (lower panel), respectively. The expression percentage of TLR2/4 on DCs stimulated with r*Ts*-Hsp70 is shown on the right. All experiments were performed three times and data are shown with mean ± SD. n = 3, ** *p* < 0.01, *** *p* < 0.001.

After being co-incubated with 5 μg/ml of r*Ts*-Hsp70 for 48 hours, both TLR2 and TLR4 were upregulated on the surface of mouse bone marrow-derived DCs compared to the cells incubated with PBS or BSA only. As positive controls, LPS (the agonist for TLR4) and Pam_3_CSK_4_ (the agonist for TLR2) also upregulated the expression of TLR4 and TLR2 at the similar level as r*Ts*-Hsp70 stimulation (Fig 2).

### The r*Ts*-Hsp70 induces the maturation of DCs through activating TLR2 and TLR4

**Fig 3:**
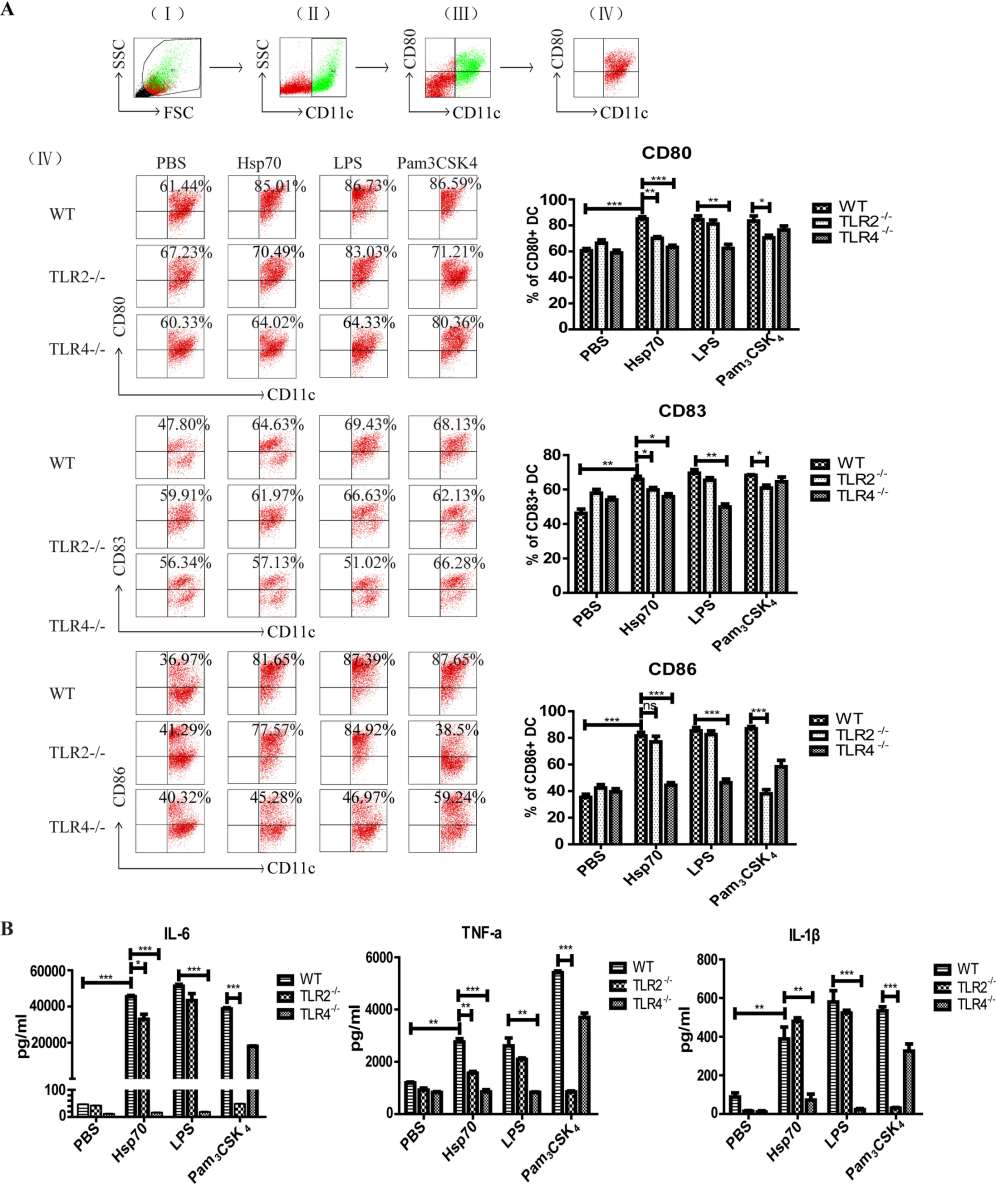
The activation of DCs by r*Ts*-Hsp70 *in vitro* was inhibited in DCs with TLR2 or TLR4 knockout. (A) Expression of co-stimulatory molecules on the surface of DCs from WT and TLR2/4^-/-^ mice stimulated by r*Ts*-Hsp70. The DCs from WT, TLR2^-/-^, TLR4^-/-^ mice were incubated with r*Ts*-Hsp70, LPS, Pam_3_CSK_4_ and PBS. Then the co-stimulatory molecules (CD80, CD83, CD86) and CD11c on the surface of DCs were detected by flow cytometry. Representative dot plots for the gating strategy: (I) gating on viable cells, (II) gating on CD11c^+^ cells, (III) selection of CD80^+^ and CD11c^+^ cells from gated R1 cells, and (IV) selection of CD80^+^ from gated CD11c^+^ cells, upper right represent percentages of double positive cells. The bar graphs on the right display the mean ± SD of every group from three experiments. (B) The secretion of cytokines by r*Ts*-Hsp70-stimulated DCs from WT or TLR2/4^-/-^ mice measured by ELISA. Quantified data are shown as mean ± SD of three separate experiments. n = 3, * *p* < 0.05, ** *p* < 0.01, *** *p* < 0.001, ns, not significant.

Our previous study has shown that r*Ts*-Hsp70 could induce the maturation of DCs i*n vitro* [15]. To determine whether r*Ts-*Hsp70 induces the maturation of DCs through activating TLR2 and/or TLR4, the DCs derived from bone marrow of WT, TLR2^-/-^ or TLR4^-/-^ mice were stimulated with r*Ts*-Hsp70. The results indicated that r*Ts*-Hsp70 strongly stimulated DCs maturation with significantly increased level of CD80, CD83 and CD86 expression on the surface of DCs from WT mice compared to DCs incubated with PBS only. The expression levels of CD80 and CD83 were significantly decreased in DCs from TLR2^-/-^ and TLR4^-/-^ mice upon stimulation with r*Ts*-Hsp70, however, the CD86 expression was only decreased in DCs from TLR4^-/-^ mice, not from TLR2^-/-^ mice. As expected in the control groups, the CD80, CD83 and CD86 expression stimulated by LPS was inhibited in DCs from TLR4^-/-^ mice, and Pam_3_CSK_4_ stimulated CD80, CD83 and CD86 expression was discharged in DCs from TLR2^-/-^ (Fig 3A).

After being stimulated with r*Ts*-Hsp70, DCs derived from WT mouse bone marrow cells secreted significant levels of IL-6, TNF-α and IL-1β compared to PBS control. The secretion of these three cytokines was highly inhibited in DCs from TLR4^-/-^ mice upon stimulation of r*Ts*-Hsp70, however, except for certain extent of inhibition of IL-6 and TNF-α there was no significant inhibition of IL-1β secretion in DCs from TLR2^-/-^ mice upon r*Ts*-Hsp70 stimulation. As controls, LPS was not able to stimulate the secretion of these three cytokines in DCs from TLR4^-/-^ mice and Pam_3_CSK_4_ could not stimulate DCs from TLR2^-/-^ mice (Fig 3B).

### r*Ts-*Hsp70-activated DCs stimulate naïve T cells

**Fig 4:**
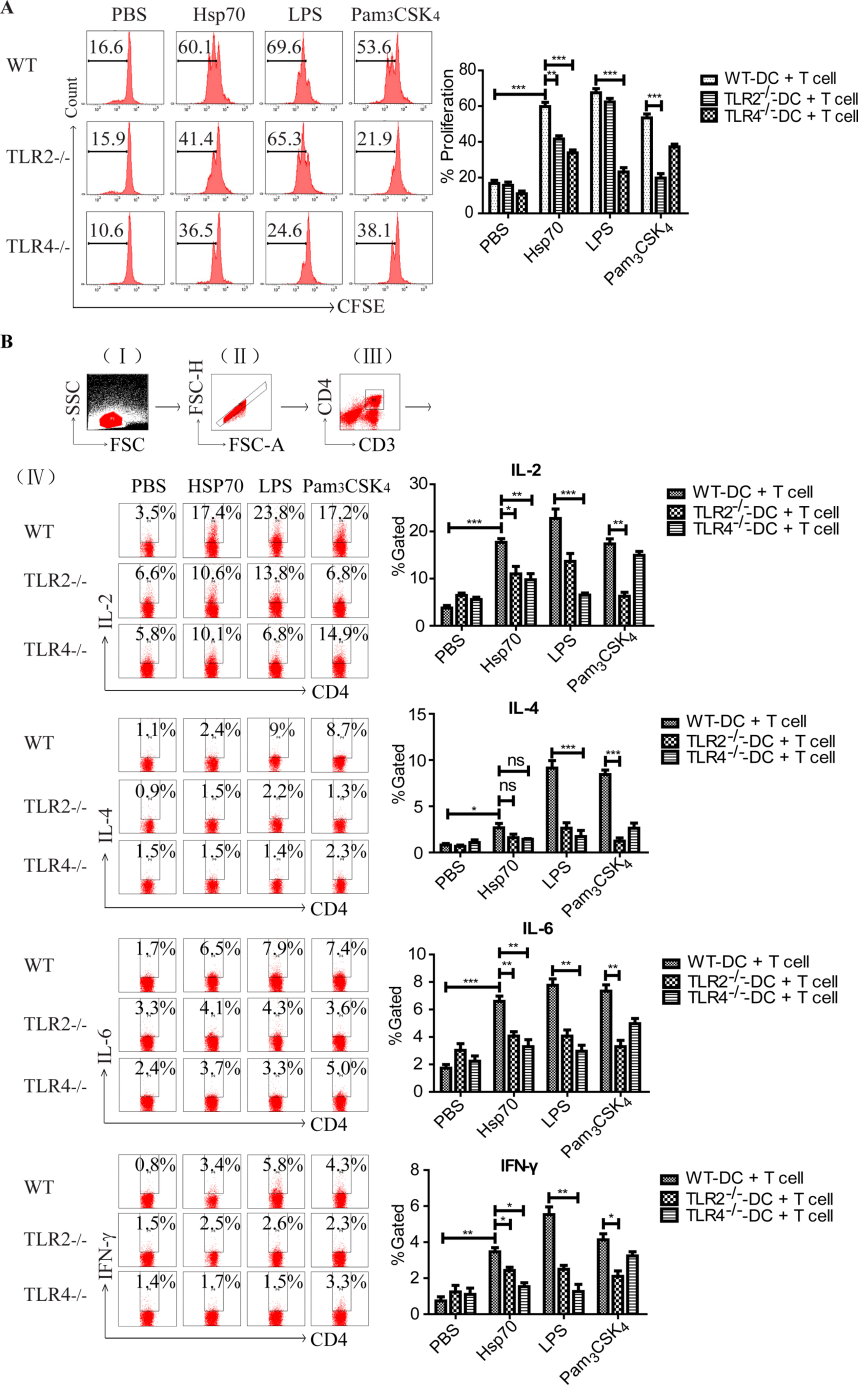
The proliferation and cytokine release of naïve splenocytes stimulated by r*Ts*-Hsp70-activated DCs. (A) The proliferation of naïve CD4^+^ T cells stimulated by r*Ts*-Hsp70-activated DCs from WT and TLR2/4^-/-^ mice determined by CFSE. The DCs from WT, TLR2^-/-^ or TLR4^-/-^ mice were pre-incubated with r*Ts*-Hsp70 or other controls including PBS, BSA, LPS and Pam_3_CSK_4_, then co-cultured with naïve mouse spleen CD4^+^ T cells. Representative histograms of the gated CD4^+^ T cells are shown in the left panel. The % of proliferation is shown as mean ± SD of three separate experiments in the right panel. n = 3, ** *p* < 0.01, *** *p* < 0.001. (B) The cytokine profile secreted by T cells stimulated by r*Ts*-Hsp70-activated DCs from WT TLR2/4^-/-^ mice was measured by intracellular cytokine staining. (I) gating on viable cells, (II) selection of non-adherent cells, (III) gating on CD3^+^ CD4^+^ T cells, and (IV) selection of IL-2^+^, IL-4^+^, IL-6^+^, IFN-γ^+^ cells from gated CD4^+^ T cells, respectively. Quantified data are shown as mean ± SD of three separate experiments. n = 3, * *p* < 0.05, ** *p* < 0.01, *** *p* < 0.001.

In our previous study, we demonstrated that r*Ts*-Hsp70 activated DCs could stimulate T cells *in vitro* [15]. To determine whether TLR2 and TLR4 are involved in this process, DCs from WT, TLR2^-/-^ or TLR4^-/-^ mice were firstly stimulated with r*Ts*-Hsp70 and then co-incubated with naïve T cells derived from splenocytes of wild type mice. The results confirmed that r*Ts*-Hsp70-primed DCs from WT mice significantly stimulated the proliferation of naïve T cells. The CD4^+^ T cell proliferation was significantly reduced when incubated with r*Ts*-Hsp70-primed DCs from TLR2^-/-^ or TLR4^-/-^ mice, especially with higher inhibition level for DCs from TLR4^-/-^ mice. In the control groups, the CD4^+^T cells proliferation was inhibited when incubated with LPS-stimulated DCs from TLR4^-/-^ mice or with Pam_3_CSK_4_-simulated DCs from TLR2^-/-^ mice (Fig 4A). The cytokine profile secreted by T cells co-incubated with activated DCs showed that IL-2, IL-6, and IFN-γ was significantly inhibited in r*Ts*-Hsp70-primed DCs from both TLR2^-/-^ or TLR4^-/-^ mice, however, no significant inhibition of IL-4 was observed in r*Ts*-Hsp70-primed DCs from both TLR2^-/-^ and TLR4^-/-^ mice (Fig 4B).

These results indicated that TLR2 and TLR4 play significant roles in the activation and maturation of DCs stimulated by r*Ts*-Hsp70, and the activated DCs can further stimulated naïve T cells.

### r*Ts*-Hsp70 induced protective immunity is compromised in TLR2/4 gene knockout mice

**Fig 5:**
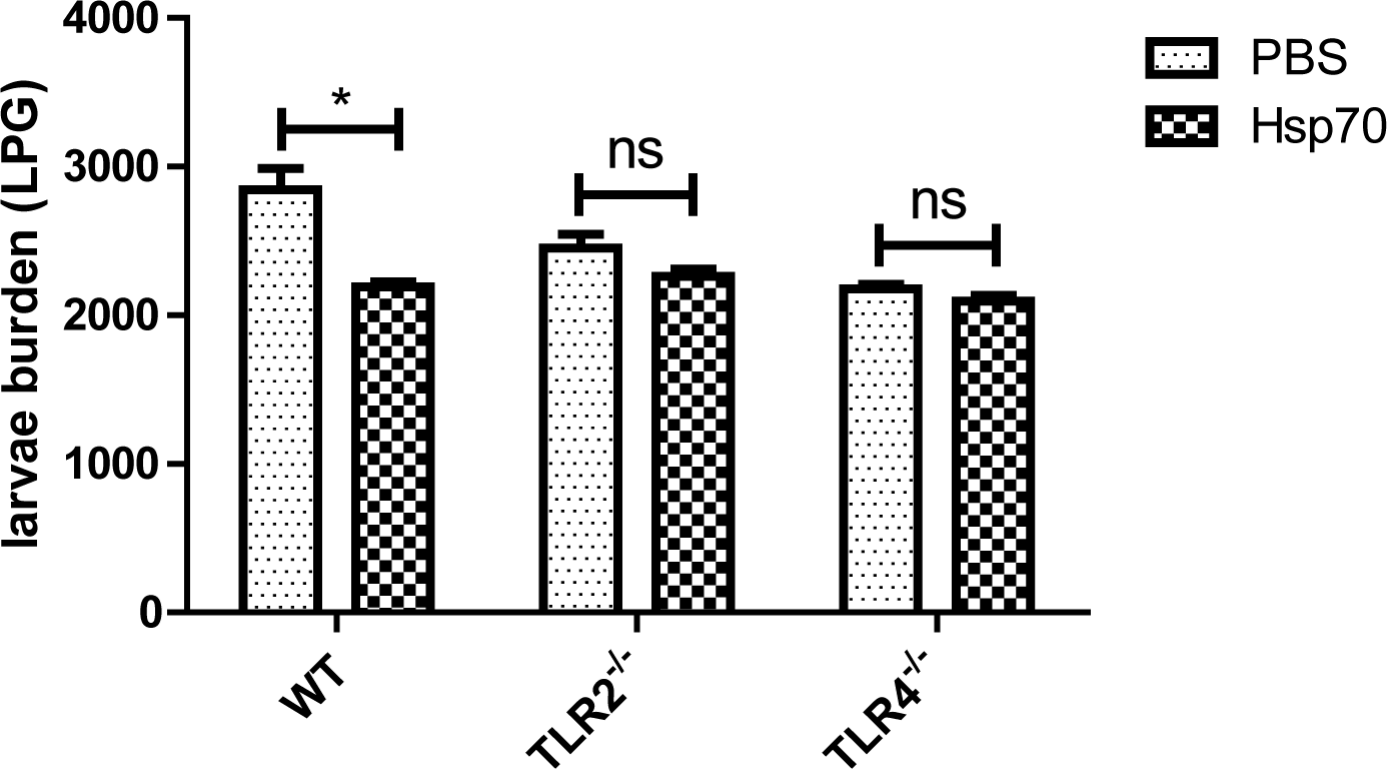
The number of larvae per gram muscle (LPG) recovered from WT, TLR2^-/-^ and TLR4^-/-^ mice immunized with r*Ts*-Hsp70 and then challenged with 500 *T*. *spiralis* larvae. Results are presented as the arithmetic mean of 10 mice per group ± SD. * *p* < 0.05, ns, not significant.

To evaluate the role of TLR2 and TLR4 involved in the r*Ts*-Hsp70 induced protective immunity *in vivo*, WT, TLR2^-/-^ and TLR4^-/-^ mice were immunized with r*Ts*-Hsp70 and then challenged with *T*. *spiralis* infective larvae. The necropsy results showed that immunization with r*Ts*-Hsp70 without adjuvant induced 22.90% ML burden reduction in WT mice compared to PBS control group with statistical difference (*p* < 0.05). The larval burden reduction was only 14.3% in immunized TLR2^-/-^ mice and 3.9% in TLR4^-/-^ mice that was not statistically significant compared to each PBS control (Fig 5). These results demonstrated that the protective immunity against *T*. *spiralis* infection induced by r*Ts*-Hsp70 is TLR2 and TLR4-dependent.

### Cellular and humoral immune response was dampened upon r*Ts*-Hsp70 immunization in TLR2/4 knockout mice

**Fig 6:**
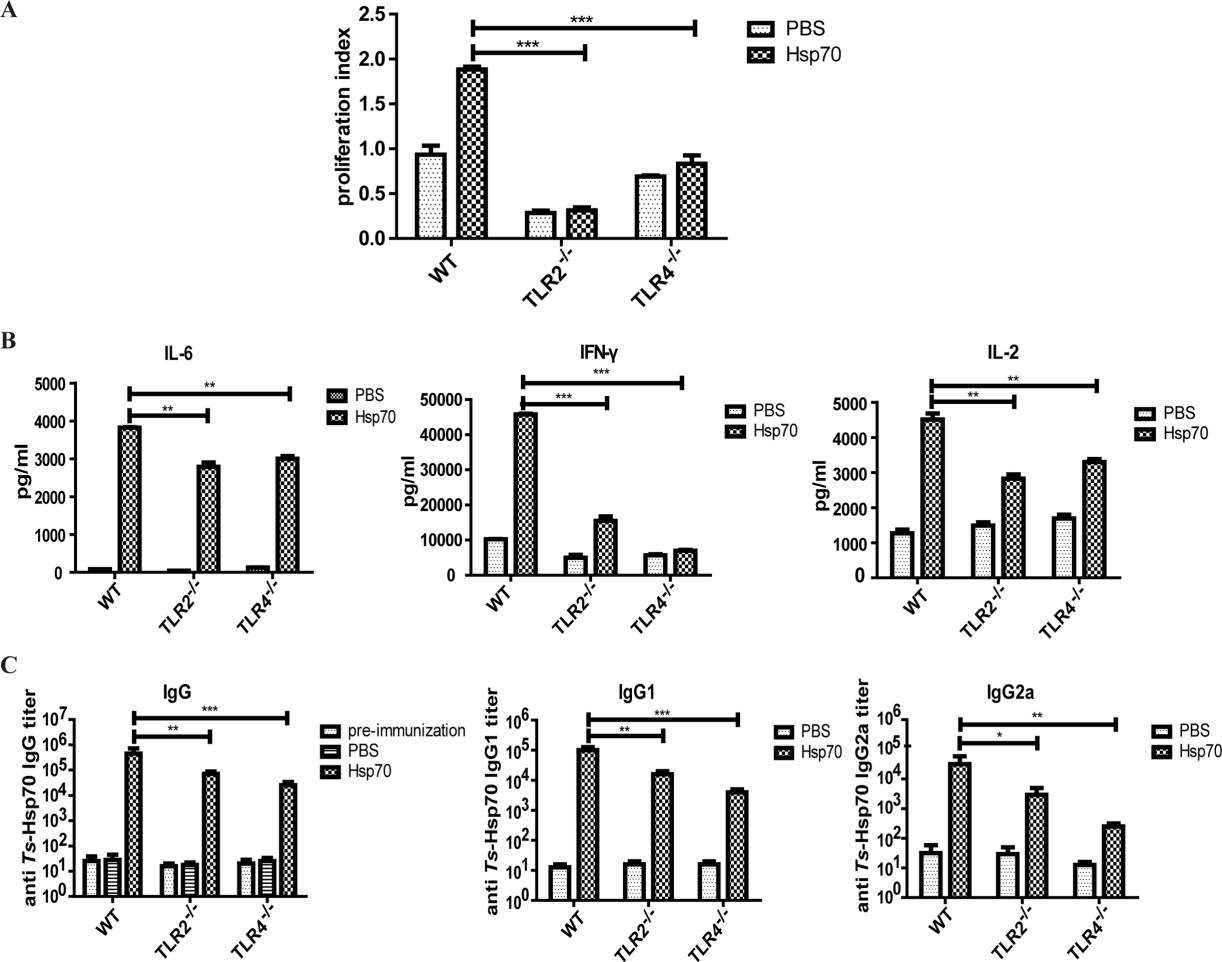
Reduced cellular and humoral antibody response induced by r*Ts*-Hsp70 in TLR2/4^-/-^ mice. (A) The proliferation of splenocytes from r*Ts*-Hsp70 immunized WT, TLR2^-/-^, TLR4^-/-^ mice upon stimulation with r*Ts*-Hsp70 was measured by MTS colorimetric assay. (B) The cytokine-secretion of splenocytes from r*Ts*-Hsp70-immunized WT, TLR2^-/-^ and TLR4^-/-^ mice upon re-stimulation of r*Ts*-Hsp70 measured by ELISA. (C) Serological anti-r*Ts*-Hsp70 IgG, IgG1 and IgG2a antibody titers against r*Ts*-Hsp70 in WT, TLR2^-/-^ and TLR4^-/-^ mice measured by ELISA. Pre-immunization control was serological anti-r*Ts*-Hsp70 IgG antibody titers of 0 days before immunization. The endpoint values are shown. Quantified data are shown as mean ± SD of three separate experiments. n = 10, * *p* < 0.05, ** *p* < 0.01, *** *p*< 0.001.

To determine the role of TLR2 and TLR4 in the immune response against r*Ts*-Hsp70, the splenocytes were isolated from immunized mice and re-stimulated with r*Ts*-Hsp70. The proliferation of splenocytes both from r*Ts*-Hsp70-immunized TLR2^-/-^ and TLR4^-/-^ mice was significantly reduced compared with that of r*Ts*-Hsp70-immunized WT mice (Fig 6A). The cytokine profile also showed that the level IL-2, IFN-γ, and IL-6 secreted by r*Ts*-Hsp70 re-stimulated splenocytes from Hsp70-immunized TLR2^-/-^ and TLR4^-/-^ mice was significantly reduced compared with those secreted by r*Ts*-Hsp70-immunized WT mice splenocytes (Fig 6B). The level of IL-4 secreted by splenocytes from r*Ts*-Hsp70 immunized WT, TLR2^-/-^ and TLR4^-/-^ mice was undetectable. The reduction of splenocytes proliferation and cytokines secretion suggested that cellular immune response against r*Ts*-Hsp70 was compromised in TLR2 and TLR4 defected mice *in vivo*.

Antibody responses in r*Ts*-Hsp70 immunized mice also showed that anti-r*Ts*-Hsp70 specific IgG, IgG1, IgG2a antibody titers were significantly decreased in sera of both TLR2^-/-^ and TLR4^-/-^ mice immunized with r*Ts*-Hsp70 compared with that detected in sera of immunized WT mice. The decreased antibody level was more significant in TLR4^-/-^ mice than in TLR2^-/-^ mice (Fig 6C). The results suggested that the humoral immune response against r*Ts*-Hsp70 were compromised in TLR2^-/-^ and TLR4^-/-^ mice.

All the above data demonstrated that TLR2 and TLR4 play important roles in the cellular and humoral immune response induced by r*Ts*-Hsp70 *in vivo*.

## Discussion

TLRs are important members of PRR family expressed on the surface of DCs, macrophages or other antigen presenting cells [33, 34]. Different TLRs recognizes distinct pathogens including bacteria, fungi, viruses and parasites [35, 36]. TLRs stimulation signaling simultaneously induces maturation of DCs [37, 38]. TLRs not only act as innate sensor but also shape and bridge innate and adaptive immune responses. They have the unique capacity to sense the initial infection and are the most potent inducers of the inflammatory responses [39]. Many researches have explored the role of TLR2 and TLR4 in the induction of host immunity against major parasitic diseases such as leishmaniasis [40], malaria [41], trypanosomiasis [42], filariasis [43] and schistosomiasis [44].These researches reveal that stimulation of host immune response with TLR2 and TLR4 agonist can be the option of choice to treat such parasitic diseases in future.

Heat shock proteins have been identified as effective vaccine candidates [45], targeting and activating DCs [46]. In particular, previous research showed that r*Ts*-Hsp70 induced protective immunity against *T*. *spiralis* infection through activating host DCs [15], however, the protective mechanism behind this observation remains unclear.

In this study, the role of TLR2 and TLR4 played in r*Ts*-Hsp70 activating DCs was investigated. Our results demonstrated that r*Ts*-Hsp70 upregulated both TLR2 and TLR4 expression on the surface of DCs *in vitro*. The upregulated level was similar as that stimulated by TLR4 agonist LPS, or TLR2 agonist Pam_3_CSK_4_. We also determine that r*Ts*-Hsp70 actually binds to TLR2 or TLR4 on the surface of DCs. This direct binding was inhibited by pre-adding TLR2 or TLR4 blocking antibody, and greatly reduced in DCs derived from mice with TLR2 or TLR4 knockout, further confirming that r*Ts*-Hsp70 binds to the surface of DCs through TLR2 and TLR4. As we have shown in our previous study, in this study we also demonstrated that r*Ts*-Hsp70 strongly induced bone marrow-derived DCs maturation characterized by the high level expression of co-stimulator molecules CD80, CD83 and CD86 and secretion of IL-6, TNF-α and IL-1β, the typical biomarkers of DCs maturation [15], and these stimulated maturation markers were decreased in DCs derived from TLR4 and TLR2 knockout mouse bone marrow cells, indicating that r*Ts*-Hsp70 stimulates DCs through activating TLR2 and TLR4 on the surface of DCs. Most of evidences showed that r*Ts*-Hsp70 activated both TLR2 and TLR4 on DCs, however, the r*Ts*-Hsp70 induced DCs maturation was dramatically reduced or demolished in those derived from TLR4 knockout mice, but reduced at a less level or not reduced (such as CD86, IL-1β) in DCs derived from TLR2 knockout mice, indicating r*Ts*-Hsp70 may activate DCs more through TLR4 than through TLR2. However, the underlying mechanisms need to be further studied.

To determine whether TLR2 and TLR4 involved in activation of naïve T lymphocytes induced by r*Ts*-Hsp70-stimulated DCs, r*Ts*-Hsp70 activated DCs derived from WT or TLR2^-/-^ and TLR4^-/-^ mice were co-incubated with CD4^+^ T cells from naïve normal mice. The co-stimulation results demonstrated that the CD4^+^ T cells proliferation induced by r*Ts*-Hsp70-primed DCs from TLR2^-/-^ and TLR4^-/-^ mice was decreased compared with that from WT mice, and the reduced CD4^+^ T cells proliferation was more obvious in DCs without TLR4 than DCs without TLR2. It is consistent with the less reduced level of IL-2, IL-6 and INF-γ secreted by T cells co-incubated with DCs without TLR2 than with DCs without TLR4. The results further confirm that r*Ts*-Hsp70 activated DCs mostly through TLR4 and less through TLR2, that can be passed to further activate downstream acting T cells, possibly through matured DCs secreted cytokines such as IL-6, TNF-α and IL-1β.

To evaluate the role of TLR2 and TLR4 played in protective immunity against *T*. *spiralis* infection induced by r*Ts*-Hsp70 *in vivo*, WT, TLR2^-/-^ and TLR4^-/-^ mice were immunized with r*Ts*-Hsp70 alone without adjuvant and then challenged with *T*. *spiralis* infective larvae. The immunization results showed that the WT mice immunized with r*Ts*-Hsp70 induced 22.9% muscle larva reduction with statistical significance compared with mice received PBS only, however, the TLR2^-/-^ and TLR4^-/-^ mice immunized with the same amount of r*Ts*-Hsp70 elicited significantly less muscle larva reduction (14.30% and 3.90%, respectively), indicating both TLR2 and TLR4 are involved in the protective immunity induced by r*Ts*-Hsp70. The reduced protection in TLR2 and TLR4 knockout mice is related to the significant lower anti-r*Ts*-Hsp70 IgG, IgG1 and IgG2a titer, less T cells proliferation and lower levels of cytokines (IL-6, IL-4, INF-γ and IL-2) compared to WT mice, indicating the impaired immunity responses in TLR2 and TLR4 knockout mice that compromise the r*Ts*-Hsp70 induced protective immunity. It is generally believed that the Th2 immune is essential for protective immunity against helminth infection [47–49]. The humoral responses play significant roles in controlling *Trichinella* infection by participating in entrapping and expulsing infective larvae, reducing adult worms fecundity and killing newborn larvae [50]. It has also been demonstrated that the cellular immune response is involved in reducing worms in the intestinal track and muscle during infection of *T*. *spiralis* [51–53]. All the data suggested that both cellular and humoral immune response induced by r*Ts*-Hsp70 were reduced in TLR2 and TLR4 defected mice *in vivo*, which caused less ML burden reduction in r*Ts*-Hsp70-immunized TLR2^-/-^ mice and TLR4^-/-^ mice than that in r*Ts*-Hsp70-immunized WT mice. It is well known that different microbial compounds or antigens activate the maturation of DCs into Th1 cell-promoting (DC1) or Th2 cell-promoting (DC2) effector cells to polarize Th1 and Th2 T-cell responses respectively [54]. Some Gram-positive or negative bacteria stimulated TLR2 on DCs to differentiate into a Th1-promoting phenotype. Most of helminth-derived extracts promote Th2 responses through activating TLR4 on DCs [26], however, some helminth-derived antigens such as *Schistosoma mansoni* derived phosphatidylserine activated TLR2 on DCs, but drive Th2 responses and the development of T regulatory cells in the presence of TLR4 agonist LPS through different signal pathway [24]. The polarization of Th1 or Th2 responses is a result of antigen-dependent combination of TLR2 and TLR4 stimulation through distinct signaling pathway [25]. *Trichinella*-expressed *Ts*-Hsp70 stimulates both TLR2 and TLR4 on DCs to induce mixed Th1 and Th2 responses that may be related to the protective immunity.

TLR2 and TLR4 are expressed on the antigen presenting cells, including DCs and macrophages. The reduced protective immunity induced by r*Ts*-Hsp70 immunization in mice with TLR2 and TLR4 knockout possibly attribute to the less activation of DCs through TLR2 and TLR4 activation pathway. However, we cannot exclude that other antigen presenting cells such as macrophages that express TLR2 and TLR4 may also have been weakened for their ability to process antigen and initiate immune response. Moreover, there may be other possible pathways manipulated by *Ts*-Hsp70 which may play roles of the immune protection, such as the adjuvant effect of Hsps. By binding with the PRRS on APCs, Hsps activate the innate immune response and provide the host with a rapid mechanism for detecting infection by pathogens and initiates adaptive immunity [55].

The lower muscle larva reduction rate observed in WT mice immunized with r*Ts*-Hsp70 in this study (22.9%) compared to our previous immunization study (37%) [14] is due to the lack of adjuvant used in this study. Most of adjuvant boosts immunogenicity of immunized antigen more or less through activating TLRs or other antigen activation pathway [56]. In order to exclude other TLR activation pathway in this study, we directly immunize mice with plain r*Ts*-Hsp70 without being formulated with any adjuvant.

In conclusion, our results demonstrated that r*Ts*-Hsp70 activated DCs and initiated their maturation through directly binding to TLR2 and TLR4. TLR2 and TLR4 play vital roles in r*Ts*-Hsp70 activating DCs and immune response against *T*. *spiralis* infection, however, we can not exclude other PRR activation pathways in APCs which needs to be further investigated. This study reveals the activation pathway of r*Ts*-Hsp70-induced maturation of DCs and helps us better understand the process of r*Ts*-Hsp70 inducing protective immunity against *T*. *spiralis* infection *in vivo*, therefore, provides a better approach to rationalize and design the r*Ts*-Hsp70 as a potent vaccine for controlling trichinellosis.
